# Cerebrospinal fluid biomarkers for cerebral amyloid angiopathy

**DOI:** 10.1093/braincomms/fcad159

**Published:** 2023-05-19

**Authors:** Jochen A Sembill, Christoph Lusse, Mathias Linnerbauer, Maximilian I Sprügel, Anne Mrochen, Michael Knott, Tobias Engelhorn, Manuel Alexander Schmidt, Arnd Doerfler, Timo Jan Oberstein, Juan Manuel Maler, Johannes Kornhuber, Piotr Lewczuk, Veit Rothhammer, Stefan Schwab, Joji B Kuramatsu

**Affiliations:** Department of Neurology, University Hospital Erlangen, and Friedrich-Alexander-Universität Erlangen-Nürnberg, Erlangen 91054, Germany; Department of Neurology, University Hospital Erlangen, and Friedrich-Alexander-Universität Erlangen-Nürnberg, Erlangen 91054, Germany; Department of Neurology, University Hospital Erlangen, and Friedrich-Alexander-Universität Erlangen-Nürnberg, Erlangen 91054, Germany; Department of Neurology, University Hospital Erlangen, and Friedrich-Alexander-Universität Erlangen-Nürnberg, Erlangen 91054, Germany; Department of Neurology, University Hospital Erlangen, and Friedrich-Alexander-Universität Erlangen-Nürnberg, Erlangen 91054, Germany; Department of Neuroradiology, University Hospital Erlangen, and Friedrich-Alexander-Universität Erlangen-Nürnberg, Erlangen 91054, Germany; Department of Neuroradiology, University Hospital Erlangen, and Friedrich-Alexander-Universität Erlangen-Nürnberg, Erlangen 91054, Germany; Department of Neuroradiology, University Hospital Erlangen, and Friedrich-Alexander-Universität Erlangen-Nürnberg, Erlangen 91054, Germany; Department of Neuroradiology, University Hospital Erlangen, and Friedrich-Alexander-Universität Erlangen-Nürnberg, Erlangen 91054, Germany; Department of Psychiatry and Psychotherapy, University Hospital Erlangen, and Friedrich-Alexander-Universität Erlangen-Nürnberg, 91054 Erlangen, Germany; Department of Psychiatry and Psychotherapy, University Hospital Erlangen, and Friedrich-Alexander-Universität Erlangen-Nürnberg, 91054 Erlangen, Germany; Department of Psychiatry and Psychotherapy, University Hospital Erlangen, and Friedrich-Alexander-Universität Erlangen-Nürnberg, 91054 Erlangen, Germany; Department of Psychiatry and Psychotherapy, University Hospital Erlangen, and Friedrich-Alexander-Universität Erlangen-Nürnberg, 91054 Erlangen, Germany; Department of Neurodegeneration Diagnostics, Medical University of Bialystok, and Department of Biochemical Diagnostics, University Hospital of Bialystok, 15-090 Bialystok, Poland; Department of Neurology, University Hospital Erlangen, and Friedrich-Alexander-Universität Erlangen-Nürnberg, Erlangen 91054, Germany; Department of Neurology, University Hospital Erlangen, and Friedrich-Alexander-Universität Erlangen-Nürnberg, Erlangen 91054, Germany; Department of Neurology, University Hospital Erlangen, and Friedrich-Alexander-Universität Erlangen-Nürnberg, Erlangen 91054, Germany

**Keywords:** cerebral amyloid angiopathy, small vessel disease, cerebrospinal fluid, biomarker, dementia

## Abstract

Integrating cerebrospinal fluid-biomarkers into diagnostic workup of patients with sporadic cerebral amyloid angiopathy may support early and correct identification. We aimed to identify and validate clinical- and cerebrospinal fluid-biomarkers for *in vivo* diagnosis of cerebral amyloid angiopathy. This observational cohort study screened 2795 consecutive patients admitted for cognitive complaints to the academic departments of neurology and psychiatry over a 10-year period (2009–2018). We included 372 patients with available hemosiderin-sensitive MR imaging and cerebrospinal fluid-based neurochemical dementia diagnostics, i.e. Aβ40, Aβ42, *t*-tau, *p*-tau. We investigated the association of clinical- and cerebrospinal fluid-biomarkers with the MRI-based diagnosis of cerebral amyloid angiopathy, applying confounder-adjusted modelling, receiver operating characteristic and unsupervised cluster analyses. We identified 67 patients with cerebral amyloid angiopathy, 76 patients with Alzheimer’s disease, 75 patients with mild cognitive impairment due to Alzheimer’s disease, 76 patients with mild cognitive impairment with unlikely Alzheimer’s disease and 78 healthy controls. Patients with cerebral amyloid angiopathy showed a specific cerebrospinal fluid pattern: average concentration of Aß40 [13 792 pg/ml (10 081–18 063)] was decreased compared to all controls (*P* < 0.05); Aß42 [634 pg/ml (492–834)] was comparable to Alzheimer’s disease and mild cognitive impairment due to Alzheimer’s disease (*P* = 0.10, *P* = 0.93) but decreased compared to mild cognitive impairment and healthy controls (both *P* < 0.001); *p*-tau [67.3 pg/ml (42.9–91.9)] and *t*-tau [468 pg/ml (275–698)] were decreased compared to Alzheimer’s disease (*P* < 0.001, *P* = 0.001) and mild cognitive impairment due to Alzheimer’s disease (*P* = 0.001, *P* = 0.07), but elevated compared to mild cognitive impairment and healthy controls (both *P* < 0.001). Multivariate modelling validated independent clinical association of cerebral amyloid angiopathy with older age [odds-ratio: 1.06, 95% confidence interval (1.02–1.10), *P* < 0.01], prior lobar intracerebral haemorrhage [14.00 (2.64–74.19), *P* < 0.01], prior ischaemic stroke [3.36 (1.58–7.11), *P* < 0.01], transient focal neurologic episodes (TFNEs) [4.19 (1.06–16.64), *P* = 0.04] and gait disturbance [2.82 (1.11–7.15), *P* = 0.03]. For cerebrospinal fluid-biomarkers per 1 pg/ml, both lower Aß40 [0.9999 (0.9998–1.0000), *P* < 0.01] and lower Aß42 levels [0.9989 (0.9980–0.9998), *P* = 0.01] provided an independent association with cerebral amyloid angiopathy controlled for all aforementioned clinical confounders. Both amyloid biomarkers showed good discrimination for diagnosis of cerebral amyloid angiopathy among adjusted receiver operating characteristic analyses (area under the receiver operating characteristic curves, Aß40: 0.80 (0.73–0.86), *P* < 0.001; Aß42: 0.81 (0.75–0.88), *P* < 0.001). Unsupervised Euclidian clustering of all cerebrospinal fluid-biomarker-profiles resulted in distinct segregation of cerebral amyloid angiopathy patients from all controls. Together, we demonstrate that a distinctive set of cerebrospinal fluid-biomarkers effectively differentiate cerebral amyloid angiopathy patients from patients with Alzheimer’s disease, mild cognitive impairment with or without underlying Alzheimer’s disease, and healthy controls. Integrating our findings into a multiparametric approach may facilitate diagnosing cerebral amyloid angiopathy, and may aid clinical decision-making, but warrants future prospective validation.

## Teaser

Sembill *et al*. reports observational data from 372 patients showing that cerebrospinal fluid-based neurochemical dementia diagnostics, specifically decreased ß-amyloid 40, independently distinguish patients with cerebral amyloid angiopathy from those with Alzheimer’s disease, mild cognitive impairment and healthy controls.

## Graphical Abstract

**Graphical abstract:**
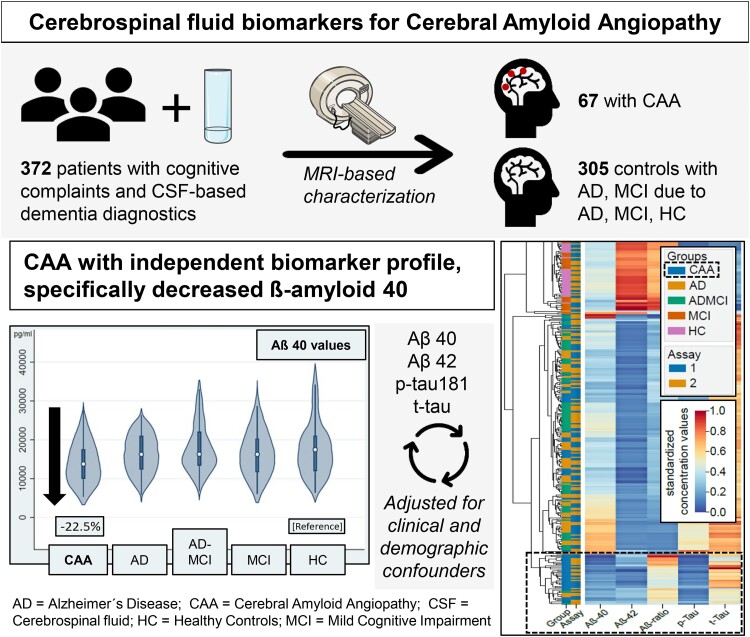

## Introduction

Sporadic cerebral amyloid angiopathy (CAA) represents the most common cerebral small vessel disease in the elderly, which is clinically associated with a higher risk for haemorrhagic and ischaemic stroke, as well as dementia. Patients further frequently complain of gait disturbances, mood disorders, and the occurrence of transient focal neurologic episodes (TFNE, ‘amyloid spells’).^1-3^ Pathophysiologically, progressive deposition of β-amyloid (Aβ) into walls of cortical and leptomeningeal small arterioles leads to disturbance of neurovascular units, activation of inflammatory cascades, and impaired cerebral autoregulation.^1,2^ Upon neuroimaging specific cortical and subcortical lesions characterize the *in vivo* diagnosis for CAA, established by the MRI-based modified Boston criteria.^1,4^ However, these structural MRI pathologies likely represent late and irreversible brain damage from continued β-amyloid accumulation.^5^ Neurochemically, soluble molecules are in constant equilibrium between interstitial fluid and cerebrospinal fluid (CSF). Hence, levels of neurochemical dementia diagnostic (NDD) biomarkers, i.e. Aβ40, Aβ42, Aβ40/Aβ42-ratio, *t*-tau, *p*-tau, bare the potential for early CAA detection—analogous to CSF-based diagnosis of Alzheimer disease at earlier stages.^5-7^ Available data remains sparse with one aggregate data meta-analysis, including 59 CAA patients in total from five cohorts, suggesting that Aβ40 concentrations—as the major isoform in CAA—may be useful to discern CAA.^5,8-10^ This finding was supported in a subsequent study of 63 patients with CAA,^11^ whereas another analysis of 31 CAA patients contrarily documented that amyloid biomarkers were not helpful in distinguishing CAA from patients.^12^

For the present study, in a first step, we investigated crude levels of CSF-NDD-biomarkers in patients with CAA identified by MRI-based modified Boston criteria and compared them to those of patients with Alzheimer’s disease, mild cognitive impairment (MCI) due to Alzheimer’s disease, MCI with unlikely Alzheimer’s disease, and to healthy controls (HC). In a second step, we identified independent associations with the *in vivo* diagnosis of CAA among clinical- and CSF-biomarkers. Finally, we validated the discriminative ability of these CSF-biomarker profiles following confounder adjustments by using receiver operating characteristic (ROC) analyses and Euclidian clustering.

## Material and methods

### Study design and participants

**Figure 1:**
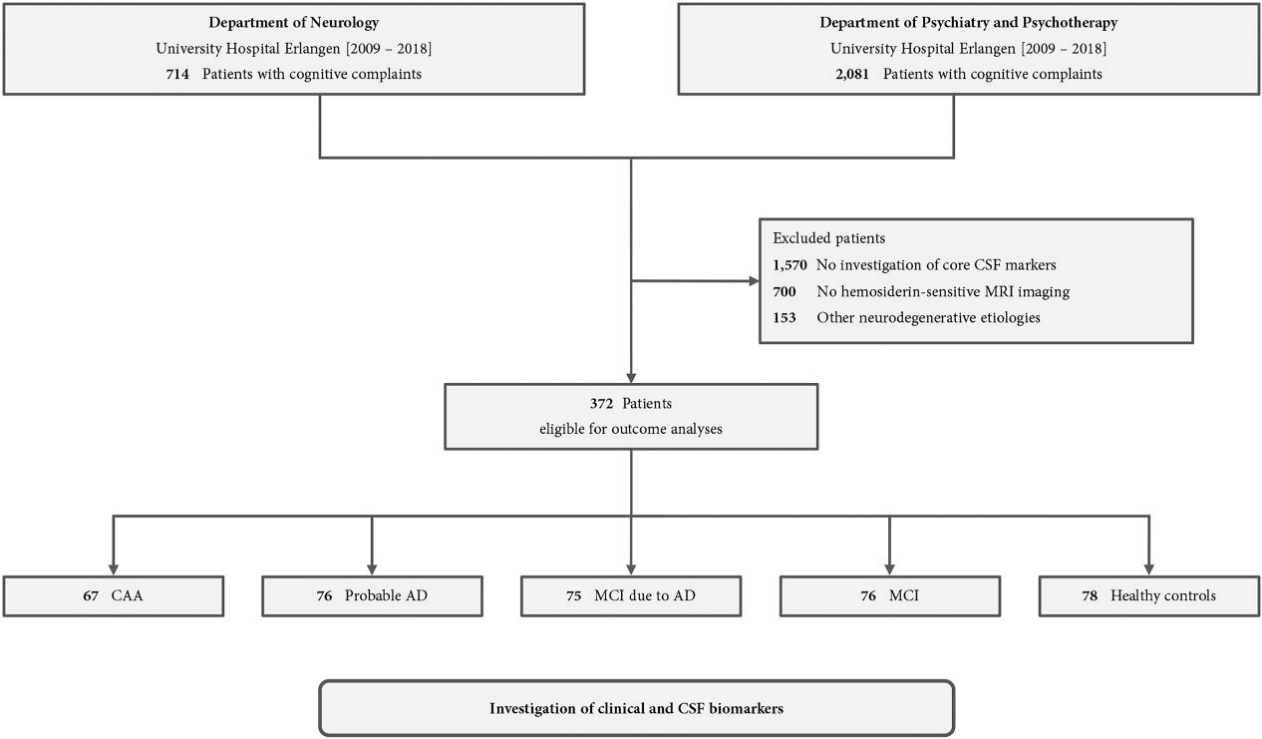
**Study flowchart.** We screened 2795 consecutive patients with cognitive complaints from the departments of neurology or psychiatry and psychotherapy of the University Hospital Erlangen from 2009 until 2018. There were 372 patients with either CAA, Alzheimer’s disease, MCI due to Alzheimer’s disease, MCI with unlikely Alzheimer’s disease, or without neurological or psychiatric disease primary related to amyloid deposition and normal cognitive tests results (healthy controls), and available MRI and CSF biomarkers eligible for outcome analyses. Abbreviations: CAA, Cerebral amyloid angiopathy; CSF, Cerebrospinal fluid; MCI, Mild cognitive impairment

The BALANCING (cereBrospinAl fLuid biomArkers iN Cerebral amyloId aNGiopathy) cohort study screened 2795 consecutive patients admitted for diagnostic evaluation of cognitive complaints to the Department of Neurology (*n* = 714) or to the Department of Psychiatry and Psychotherapy (*n* = 2081) at the University Hospital Erlangen from January 2009 until December 2018 (for study flowchart see Fig. 1). After the exclusion of patients (*n* = 1570) without lumbar puncture for assessment of CSF-NDD-biomarkers, patients (*n* = 700) without hemosiderin-sensitive MRI imaging performed within a 6-month time-frame since lumbar puncture, and patients (*n* = 153) with other causal neurodegenerative diseases, 372 patients remained for retrospective analysis. We investigated 67 patients with either possible or probable CAA according to modified Boston criteria,^4^ 76 patients with probable Alzheimer’s disease and 75 patients with MCI due to Alzheimer’s disease according to the diagnostic guidelines for Alzheimer’s disease by the National Institute on Aging and the Alzheimer’s Association,^13^ 76 patients with MCI with unlikely Alzheimer’s disease,^14^ and 78 patients without any neurological or psychiatric disease primary related to amyloid deposition and with normal cognitive tests results [referred to as ‘healthy controls (HC)’^5,9^]. The ethics committee of the University Erlangen-Nuremberg approved the study protocol (Nr. 20_21 Bc) and centrally granted a waiver of informed consent.

### Data acquisition

We retrospectively obtained baseline characteristics (demographics, medical history, comorbidities, neuropsychological and clinical symptoms) through a review of patients’ medical charts and prospective databases. The level of education was categorized as low, intermediate or high according to the 2011 International Standard Classification of Education.^15^ We classified TFNE according to their clinical phenomenology as either predominantly positive or predominantly negative and assessed their duration, frequency and stereotype presentation, i.e. recurrent episodes similar or identical to initial appearance.^16^ All patients were evaluated for competing causes, specifically transient ischaemic attack, seizures and migraine, and TFNE was scored only if these investigations were not positive. Gait disturbance was defined as an individual’s subjective perception of gait difficulties, the presence of walking aids, and objective evaluation on physical examination.^17^

CSF-NDD-biomarker levels were obtained from our prospective laboratory database for Aβ40, Aβ42, Aβ40/Aβ42 ratio, *p*-tau181, and *t*-tau. CSF-biomarkers were analysed in the Laboratory for Clinical Neurochemistry and Neurochemical Dementia Diagnostics of the Department of Psychiatry, with enzyme-linked immunosorbent assay and according to the protocols of the assays vendors [IBL International, Hamburg, Germany, and Fujirebio Europe (former Innogenetics), Ghent, Belgium for Aβ1-42; The Genetics Company (Zürich, Switzerland), and IBL International for Aβx-40 and Aβ1-40, respectively; and Fujirebio for tau and p-tau181]. During the study period, the reference ranges for the NDD biomarkers were changed due to the change of the assays used, and for reasons unrelated to this study (May 2010 for Aβ42/40, and August 2014 for the remaining biomarkers). The transition of the laboratory protocols and the diagnostic-relevant reference ranges were strictly monitored and validated according to the ISO 15189 Norm. CSF-NDD-biomarkers were interpreted according to the previously validated and widely accepted Erlangen Score algorithm.^18,19^

Imaging data were independently analysed by specialized neuroradiologists (MK, PH, MS) blinded to clinical data, and consensus was achieved for discrepant cases.^20,21^ We retrospectively investigated hemosiderin-sensitive 1.5 T or 3.0 T MRI sequences, i.e. T2*-weighted gradient-echo or susceptibility-weighted imaging as well as conventional MRI sequences, i.e. T1, T2, FLAIR. Applying the modified Boston criteria 1.5, we categorized CAA patients as either possible or probable CAA according to their age, the presence and location of macro- and micro-haemorrhages and cortical superficial siderosis, as previously described.^4^

### Outcomes

We investigated (1) the CSF-NDD-biomarker profile (Aβ40, Aβ42, Aβ40/Aβ42-ratio, *p*-tau, *t*-tau) and (2) independent clinical associations in patients with CAA compared to patients with probable Alzheimer’s disease, MCI due to Alzheimer’s disease, MCI with unlikely Alzheimer’s disease, and HC. (3) We controlled CSF-biomarkers analyses for clinical confounders and assessed their discriminative ability for the identification of patients with CAA.

### Statistical analysis

All statistical tests were two-sided and we set the significance level at *α* = 0.05 using complete case analyses as appropriate.^20,22^ Results of continuous variables are expressed as average and standard deviations (SD) for normally distributed cases and as medians and interquartile ranges (IQR) for non-normally distributed cases; results of categorical variables are expressed as proportions.^23^ Inter-group comparison was performed using the Kruskal–Wallis-test, one-way analysis of variance, Pearson *χ*² test, and Fisher exact test, as appropriate.^24^ Among parameters with relevant inter-group differences (*P* < 0.1), we computed *post hoc* test using the Mann–Whitney U-test, Student’s *t*-test, Pearson *χ*²-test or Fisher exact test.^24^ Crude CSF-biomarker levels were displayed by violin plots and compared using the Kruskal–Wallis-test and Mann–Whitney U-test. To increase the transferability of our findings, due to reported interlaboratory variations of CSF biomarker levels, we decided to calculate differences of means (DoM) with 95% confidence intervals (CI) comparing the distance from mean values of each cohort to the mean of the cohort of HC (reference).^25^ To account for potential bias introduced by the two assay periods DoM was calculated for each period separately. Among sensitivity analyses, we calculated the absolute risk and standardized mean differences between CAA patients and the pooled cohort of controls. To identify an independent association with the diagnosis of CAA, we applied multivariate regression modelling including variables showing statistical significance upon sensitivity analyses. To account for the reported inter-individual differences in overall amyloid levels, we used two separate models for Aß40 and Aß42.^26^ We applied logit regression modelling including identified independent associations as well as NDD biomarker assays used to estimate mean predicted values for CSF levels, and performed inter-group comparison of predicted values as mentioned above. Among ROC analyses adjusted for identified independent associations with CAA and applied NDD biomarker assays, we assessed the discriminative ability by calculating the area under the ROC curve (AUROC), and identified the optimal empirical cut-offs for Aβ40 and Aβ42 by the method of Liu *et al*.^27^ For unsupervised analysis, we performed clustering based on the Euclidean distance of normalized (Min–Max) mean predicted values of CSF levels using the scikit-learn library (Python v3.10).^28^ Visualization was performed using the Seaborn library (Python v3.10).^29^ To analyse the relation of imaging biomarkers with CSF biomarkers, we compared imaging characteristics of patients with CAA grouped into the same superordinate CSF-NDD cluster versus those not grouped into this cluster. Statistical analyses were performed using Stata v.14.2 (StataCorp, College Station, TX, USA).

## Results

### Patient characteristics

Among 372 patients with cognitive complaints, 67 (18.0%) were diagnosed with either possible (*n* = 38) or probable CAA (*n* = 29) according to the modified Boston criteria. Strong inter-rater agreement for MRI components of the modified Boston criteria was observed (Cohen κ [95% CI] superficial siderosis: 0.88 [0.77–0.98], lobar microbleeds: 0.86 [0.79–0.93]). Compared to HC (*n* = 78) patients with CAA differed in higher age [years, 75(68–79) versus 58(54–65), *P* < 0.001], had more frequent arterial hypertension [56(83.6%) versus 49(62.8%), *P* = 0.005], more frequent prior ischaemic stroke [19(28.4%) versus 7(9.0%), *P* = 0.002] and more frequent prior intracerebral haemorrhage (ICH) [11(16.4%) versus 1(1.3%), *P* = 0.001] notably in lobar location [9(13.4%) versus 1(1.3%), *P* < 0.01]. From a clinical perspective, patients with CAA showed more frequent apraxia [4(6.0%) versus 0(0.0%), *P* < 0.001], gait disturbance [16(23.9%) versus 1(1.3%), *P* < 0.001] and had more often TFNE [8(11.9%) versus 2(2.6%), *P* = 0.04]. For detailed inter-group comparison of patient characteristics and comparison of CAA patients to patients with probable Alzheimer’s disease (*n* = 76, 20.4%), MCI due to Alzheimer’s disease (*n* = 75, 20.2%) and MCI with unlikely Alzheimer’s disease (*n* = 76, 20.4%) as well for *post hoc* tests between specific groups see Table 1, supplemental Tables 1 and 2.

**Table 1:** Inter-group comparison of patient characteristics

Characteristics	CAA (*n* = 67)	Probable Alzheimer’s disease (*n* = 76)	MCI due to Alzheimer’s disease (*n* = 75)	MCI (*n* = 76)	HC (*n* = 78)	*P*-value
Age, years, median (IQR)	75 (68–79)	72 (60–77)	72 (65–75)	68 (57–74)	58 (54–65)	<0.001
Sex, female, *n* (%)	27 (40.3%)	39 (51.3%)	37 (49.3%)	33 (43.4%)	30 (38.5%)	0.43
Prior medical history						
Intracerebral haemorrhage, *n* (%)	11 (16.4%)	0 (0.0%)	1 (1.3%)	2 (2.6%)	1 (1.3%)	<0.001
Ischaemic stroke, *n* (%)	19 (28.4%)	9 (11.8%)	1 (1.3%)	13 (17.1%)	7 (9.0%)	<0.001
Transient ischaemic attack, *n* (%)	3 (4.5%)	5 (6.6%)	0 (0.0%)	2 (2.6%)	3 (3.8%)	0.20
Subarachnoid haemorrhage, *n* (%)	2 (3.0%)	0 (0.0%)	0 (0.0%)	2 (2.6%)	0 (0.0%)	0.16
Arterial hypertension, *n* (%)	56 (83.6%)	51 (67.1%)	48 (64.0%)	53 (69.7%)	49 (62.8%)	0.06
Diabetes mellitus, *n* (%)	17 (25.4%)	8 (10.5%)	10 (13.3%)	9 (11.8%)	11 (14.1%)	0.10
Prior medication						
Antiplatelet therapy, *n* (%)	27 (40.3%)	22 (28.9%)	29 (38.7%)	23 (30.3%)	19 (24.4%)	0.19
Oral anticoagulants, *n* (%)	5 (7.5%)	6 (7.9%)	1 (1.3%)	5 (6.6%)	1 (1.3%)	0.10
Statins, *n* (%)	23 (34.3%)	23 (30.3%)	28 (37.3%)	22 (28.9%)	15 (19.2%)	0.14
Clinical features						
Aggressive behaviour, *n* (%)	2 (3.0%)	5 (6.6%)	2 (2.7%)	0 (0.0%)	0 (0.0%)	0.04
Gait disturbance, *n* (%)	16 (23.9%)	5 (6.6%)	3 (4.0%)	8 (10.5%)	1 (1.3%)	<0.001
Recurrent falls, *n* (%)	9 (13.4%)	3 (3.9%)	3 (4.0%)	8 (10.5%)	4 (5.1%)	0.09
Transient focal neurologic episodes, *n* (%)	8 (11.9%)	4 (5.3%)	0 (0.0%)	2 (2.6%)	2 (2.6%)	<0.01

Compared by Kruskal–Wallis H-test, Pearson’s χ^2^-test, or the Freeman–Halton extension of the Fisher’s exact test. Abbreviations: CAA, Cerebral amyloid angiopathy; CI, confidence intervals; HC, healthy controls; IQR, Interquartile range; MCI, mild cognitive impairment. This table is an abbreviated version of the patient characteristics. For the complete table, please see supplemental table 1.

### Cerebrospinal fluid-neurochemical dementia diagnostic-biomarkers

**Figure 2:**
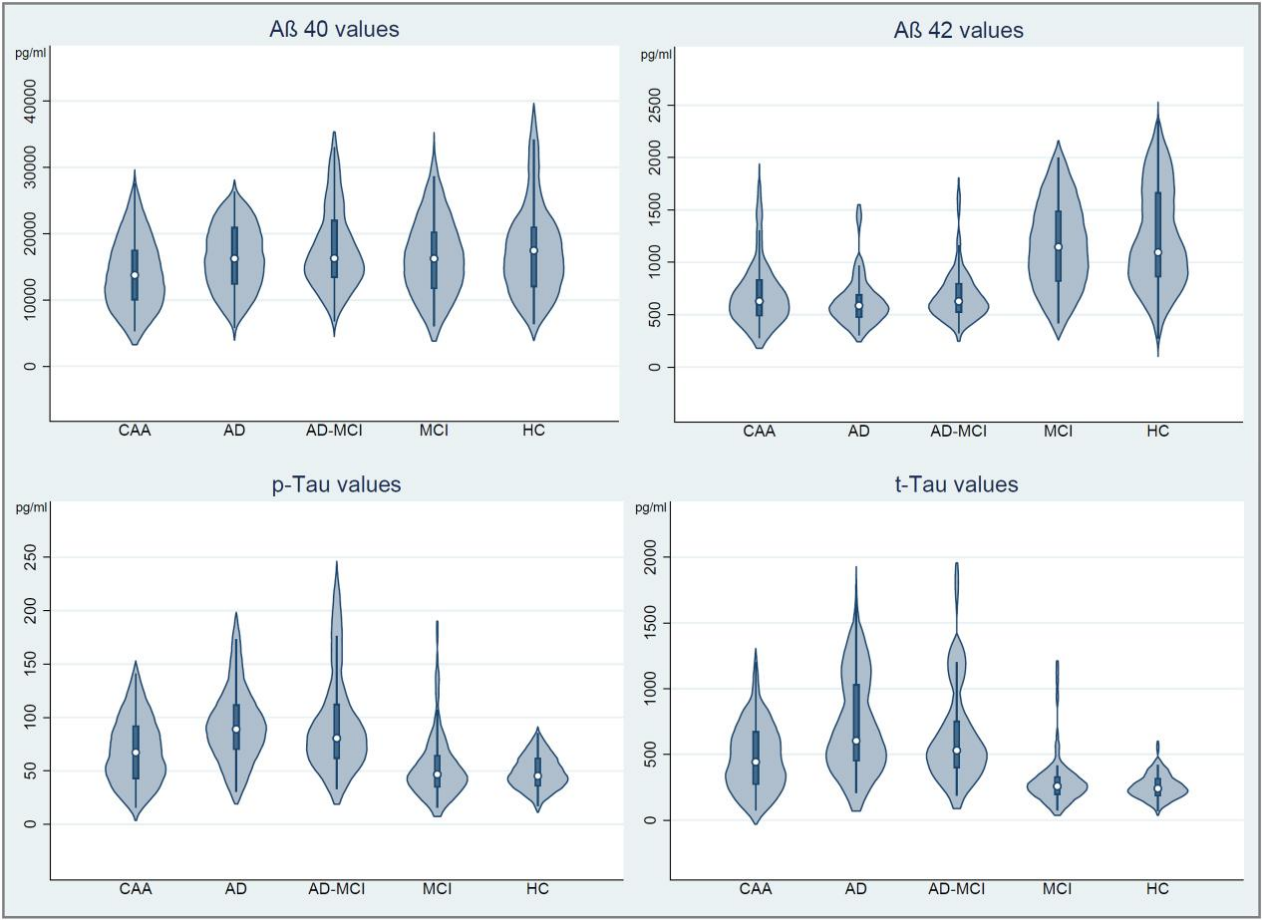
**Violin plots of core cerebrospinal fluid biomarker levels.** Graphical inter-group comparison of Aβ40, Aβ42, *p*-tau and *t*-tau using violin plots. Abbreviations: Aβ, β-amyloid; CAA, cerebral amyloid angiopathy; HC, healthy controls; MCI, mild cognitive impairment; Alzheimer’s disease-MCI, mild cognitive impairment due to Alzheimer’s disease

**Figure 3:**
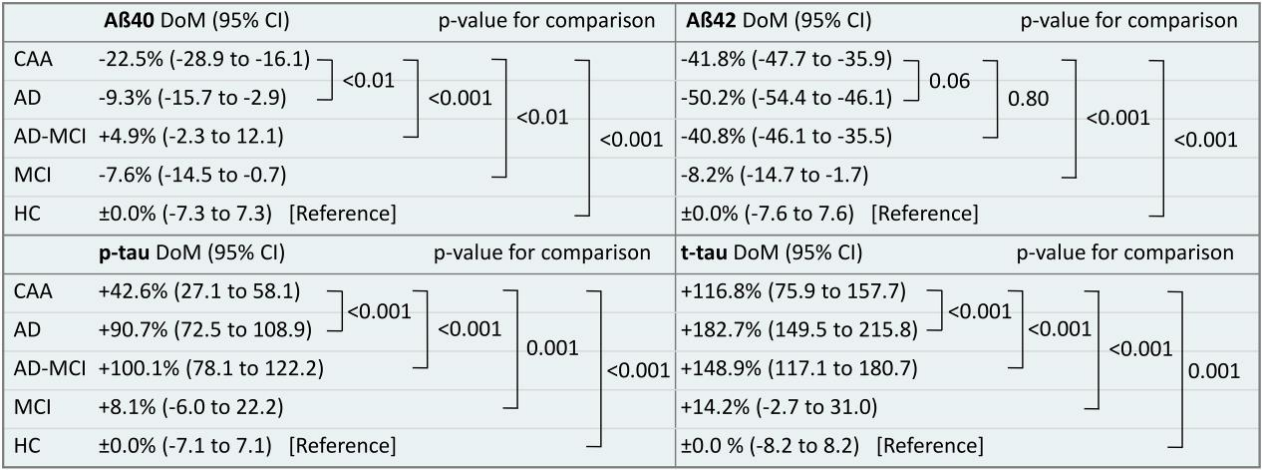
**Comparison of core cerebrospinal fluid biomarker levels.** Comparison of assay-specific differences of means (DoM) with 95% confidence intervals (CI) from crude CSF biomarker levels in relation to HC as a reference. DoM of patients with CAA was compared to all controls by Mann–Whitney U-test. Abbreviations: Aβ, β-amyloid; CAA, cerebral amyloid angiopathy; HC, Healthy controls; MCI, mild cognitive impairment; Alzheimer’s disease-MCI, mild cognitive impairment due to Alzheimer’s disease

For inter-group comparison of Aβ40, Aβ42, *p*-tau and *t*-tau see Fig. 2 displaying crude CSF levels by violin plots and Fig. 3 showing assay-specific DoM in relation to HC as a reference. Patients with CAA showed a specific CSF-NDD pattern: Aß40 [13 792 pg/ml (10 081–18 063), DoM (95% CI): −22.5% (−28.9 to −16.1)] was decreased compared to all controls, i.e. Alzheimer’s disease [16 246 pg/ml (12 410–20 906), *P* = 0.02; DoM: −9.3% (−15.7 to −2.9), *P* < 0.01], MCI due to Alzheimer’s disease [16 288 pg/ml (13 399–21 966), *P* = 0.001; DoM: +4.9% (−2.3 to 12.1), *P* < 0.001], MCI [16 241 pg/ml (11 761–20 232), *P* = 0.02; DoM: −7.6% (−14.5 to −0.7), *P* < 0.01] and HC [17 452 pg/ml (12 036–21 029), *P* < 0.01; DoM: (Reference) ± 0.0% (−7.3 to 7.3), *P* < 0.001]. Aß42 [634 pg/ml (492–834); DoM −41.8% (−47.7 to −35.9)] did not significantly differ from patients with Alzheimer’s disease [587 pg/ml (476–689), *P* = 0.10; DoM: −50.2% (−54.4 to −46.1), *P* = 0.06] or MCI due to Alzheimer’s disease [628 pg/ml (526–800), *P* = 0.93; DoM: −40.8% (−46.1 to −35.5), *P* = 0.80], but was decreased compared to patients with MCI [1148 pg/ml (822–1488), *P* < 0.001; DoM: −8.2% (−14.7 to −1.7), *P* < 0.001] or HC [1096 pg/ml (865–1662), *P* < 0.001; DoM: (reference) ± 0.0% (−7.6 to 7.6)]. The Aβ40/Aβ42-ratio was 0.047 [(0.035–0.072), DoM: −23.9% (−32.4 to −15.4)] which was higher compared to patients with Alzheimer’s disease [0.037 (0.032–0.044), *P* = 0.001; DoM: −43.5% (−48.7 to −38.4), *P* < 0.001] or MCI due to Alzheimer’s disease [0.039 (0.030–0.047), *P* < 0.01; DoM: −41.6% (−47.2 to −35.9), *P* < 0.001], but lower compared to patients with MCI [0.073 (0.059–0.083), *P* < 0.001; DoM: −1.9% (−4.5 to 8.2), *P* < 0.001] or HC [0.072 (0.059–0.083), *P* < 0.001; DoM: (reference) ± 0.0% (−5.6 to 5.6), *P* < 0.001]. *p*-tau [67.3 pg/ml (42.9–91.9), DoM: +42.6% (27.1 to 58.1)] was decreased compared to patients with Alzheimer’s disease [89.0 pg/ml (70.2–111.8), *P* < 0.001; DoM: +90.7% (72.5 to 108.9), *P* < 0.001] or MCI due to Alzheimer’s disease [80.6 pg/ml (62.1–112.0), *P* = 0.001; DoM: +100.1% (78.1 to 122.2), *P* < 0.001], and elevated compared to MCI [46.8 pg/ml (34.8–64.6), *P* < 0.001; DoM: +8.1% (−6.0 to 22.2), *P* = 0.001] or HC [45.3 pg/ml (36.1–61.7), *P* < 0.001; DoM: (reference) ± 0.0% (−7.1 to 7.1)]. Similarly, *t*-tau [468 pg/ml (275–698), DoM: 116.8% (75.9–157.7)] was decreased compared to patients with Alzheimer’s disease [604 pg/ml (454–1028), *P* = 0.001); DoM: +182.7% (149.5 to 215.8), *P* < 0.001] or MCI due to Alzheimer’s disease [531 pg/ml (401–751), *P* = 0.07; DoM: +148.9% (117.1 to 180.7), *P* < 0.001], and elevated compared to MCI [259 pg/ml (198–328), *P* < 0.001; DoM: +14.2% (−2.7 to 31.0), *P* < 0.001] or HC [242 pg/ml (187–319), *P* < 0.001; DoM: (reference) ± 0.0% (−8.2 to 8.2), *P* = 0.001]. For inter-group comparison of Aβ40/Aβ42-ratio see supplemental Fig. 2.

For results from the comparison of crude CSF levels between other groups, see supplemental Table 3. Performing intra-group comparison of CSF levels from patients with possible CAA (*n* = 38) versus probable CAA (*n* = 29), Aß40 and tau levels did not differ, but Aß42 and Aβ40/Aβ42-ratio was lower in patients with probable CAA (supplemental Table 4).

### Independent associations with cerebral amyloid angiopathy

To identify independent associations of clinical- and CSF-biomarkers with the diagnosis of CAA, we performed multivariate modelling adjusting for confounders associated with the diagnosis of CAA based on sensitivity analysis, supplemental Table 5. In particular, we included in analyses: age, arterial hypertension, diabetes mellitus, prior lobar ICH, prior ischaemic stroke and CSF amyloid levels. We calculated two separate multivariate models (1) for Aß40 (supplemental Table 6) and (2) for Aß42 (supplemental Table 7) to account for inter-individual differences in overall amyloid levels.^26^ The following variables were independently associated with CAA: older age [odds ratio (OR) 1.06, 95% CI (1.02–1.10), *P* < 0.01], prior lobar ICH [OR 14.00, CI (2.64–74.19), *P* < 0.01], prior ischaemic stroke [OR 3.36, CI (1.58–7.11), *P* < 0.01], TFNE [OR 4.19, CI (1.06–16.64), *P* = 0.04], gait disturbance [OR 2.82, CI (1.11–7.15), *P* = 0.03], both lower Aß40 [OR 0.9999, CI (0.9998–1.0000), *P* < 0.01] and lower Aß42 levels [OR 0.9989, CI (0.9980–0.9998), *P* = 0.01].

### Adjusted cerebrospinal fluid-biomarker analyses

**Figure 4:**
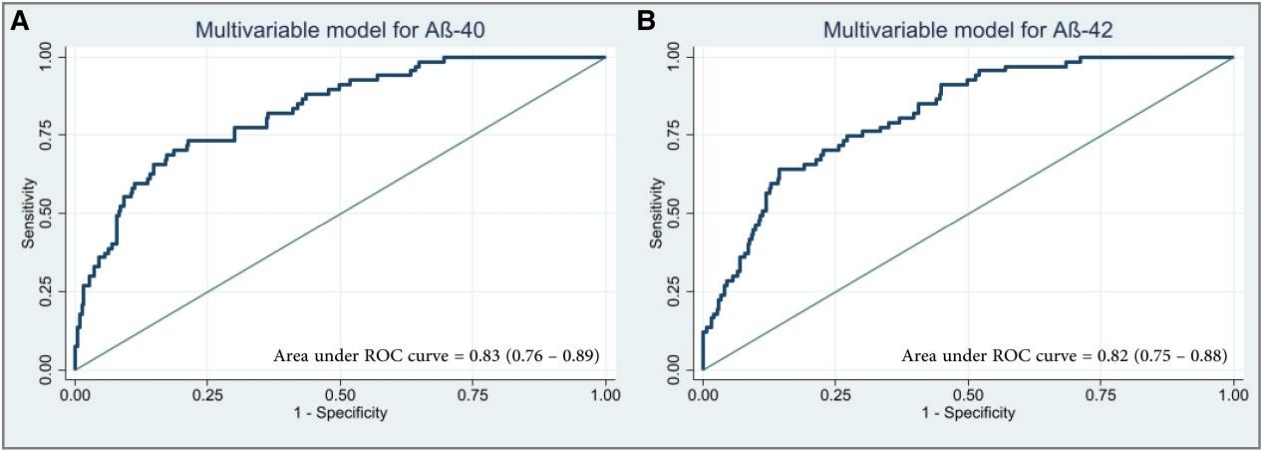
**Adjusted receiver operating characteristic curves.** Receiver operating characteristic (ROC) curves based on multivariable modelling including age, arterial hypertension, diabetes mellitus, prior lobar intracerebral haemorrhage, prior ischaemic stroke, transient focal neurological episodes, gait disturbance, *t*-tau, and β-amyloid 40 in **A** or β-amyloid 42 in **B**. Abbreviations: Aβ, β-amyloid

**Figure 5:**
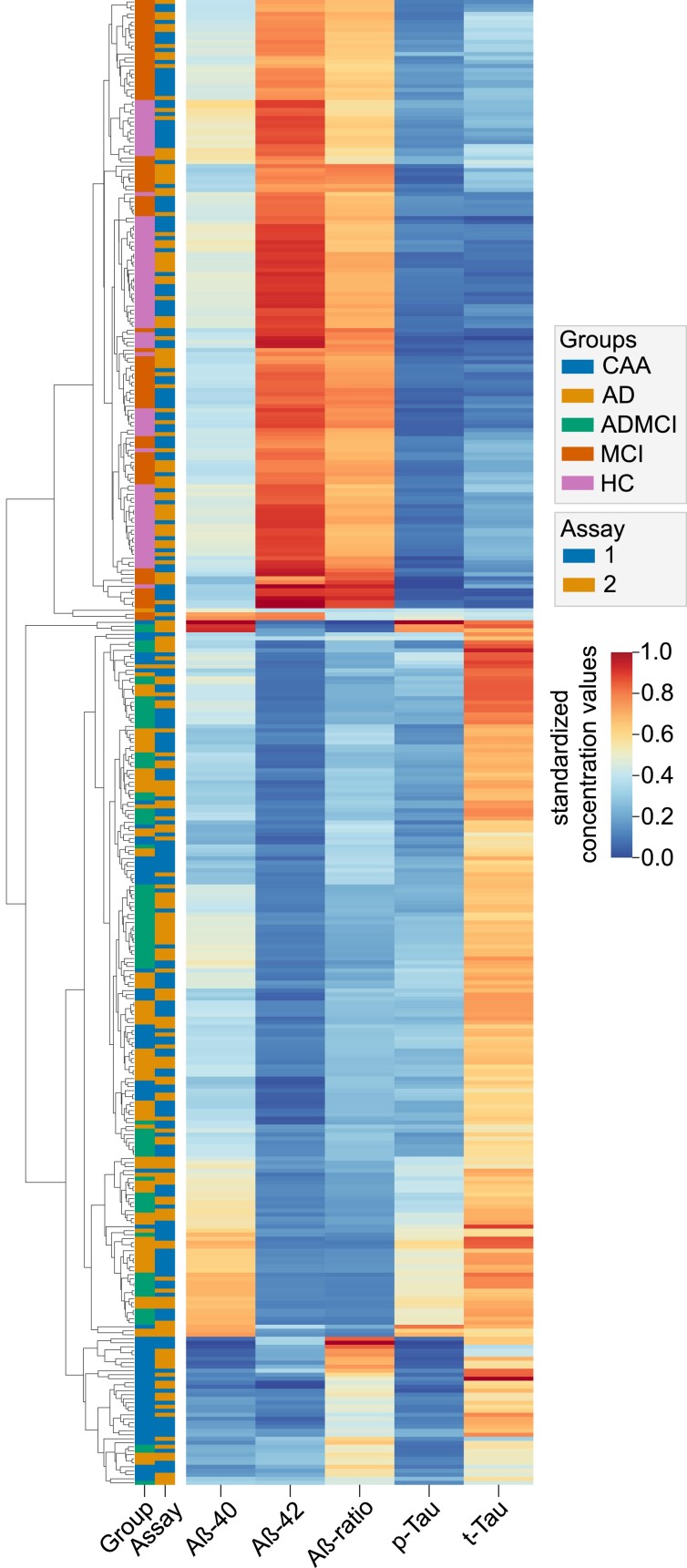
**Heatmap of cerebrospinal fluid biomarker levels analysed by Euclidean clustering.** Clustermap of normalized mean predicted CSF values based on Euclidian distance. Mean predicted values were calculated by inclusion of independent associations of clinical biomarkers with the diagnosis of CAA, i.e. age, prior lobar ICH, prior ischaemic stroke, gait disturbance and transient focal neurological episodes. Patients are colour-coded based on the underlying pathological condition and the assay used. Abbreviations: Aβ, β-amyloid; CAA, cerebral amyloid angiopathy; HC, Healthy controls; MCI, mild cognitive impairment; Alzheimer's disease-MCI, mild cognitive impairment due to Alzheimer's disease.

The specific CSF-NDD-biomarker profile of CAA patients as identified by crude analyses was confirmed upon adjusted modelling, see supplemental Fig. 3 for adjusted violin plots, supplemental Tables 8 and 9 for adjusted inter-group comparison and *post hoc* testing. Analyses were adjusted for both identified independent clinical associations as well as applied NDD biomarker assays. To investigate the discriminative ability of independently associated CSF-biomarkers with the diagnosis of CAA, we performed adjusted ROC analyses for Aß40 and Aß42. Both models showed good discrimination between diagnosis of CAA and controls [Aß40, AUROC 0.83, CI (0.76–0.89), *P* < 0.001; Aß42, AUROC 0.82, CI (0.75–0.88), *P* < 0.001], see Fig. 4A and B. The optimal cut points were 14 430 pg/ml CI (11 874–16 985) for Aß40 and 602 pg/ml CI (430–774) for Aß42. Sub-analyses showed fair discrimination of amyloid biomarkers between CAA and Alzheimer’s disease (Aß40, AUROC 0.76, CI (0.68–0.84), *P* < 0.001; Aß42, AUROC 0.75, CI (0.67–0.83), *P* < 0.001) and excellent discrimination between CAA and HC (Aß40, AUROC 0.96, CI (0.93–1.00), *P* < 0.001; Aß42, AUROC 0.95, CI (0.92–0.99), *P* < 0.001), see supplemental Fig. 4. To validate the discriminative ability, we furthermore performed unsupervised hierarchical clustering based on Euclidian distance of normalized mean predicted values (Fig. 5). Indeed, the majority of CAA patients clustered separately from patients with Alzheimer’s disease, MCI with or without underlying Alzheimer’s disease, and HC. Furthermore, Euclidian clustering revealed no segregation based on the NDD-assay used.

To further investigate the differences between CAA patients that were distinct from other patient groups and those less dissimilar based on Euclidian clustering, we analysed their MRI characteristics. In CAA patients that clustered separately from other patient groups, we observed more severe deep white matter hyperdensities [Fazekas score, 2 (1–2) versus 1 (1–2), *P* = 0.04] and more frequent neuroradiological signs of prior cerebral ischaemia [11/31 (35.5%) versus 5/36 (13.9%), *P* = 0.04], while no differences according to *in vivo* diagnostic criteria, amount or location of cerebral microbleeds or degree of superficial siderosis were detected (supplemental Table 10).

## Discussion

The present cohort study represents the largest analysis of CSF-NDD-biomarkers in patients with CAA to date. We showed that CAA is characterized by a specific CSF pattern with decreased Aβ levels, especially Aβ-40, and only marginally elevated tau proteins. Aß40 and Aß42 demonstrated good discriminative ability for diagnosis of CAA independent from identified clinical associations.

Analysing CSF-NDD-biomarkers represents an established step in diagnosing Alzheimer’s disease.^13,30^ Opposed to amyloid depositions in Alzheimer’s disease mostly consisting of the Aβ-42 isoform, in CAA both Aβ-40 and Aβ-42 were involved with Aβ-40 representing the more specific isoform.^5^ In general, parenchymal or vascular amyloid trapping is suggested to result in decreased CSF amyloid levels, whereas pathological tau aggregation is associated with neuronal loss and atrophy, correlating with increased CSF tau proteins.^8,31^ In 2018, Charidimou *et al*. performed a meta-analysis assessing CSF data from five eligible CAA patient cohorts (*n* = 59) versus HC (*n* = 94) and versus patients with Alzheimer’s disease (*n* = 158). This investigation supports the hypothesis that a specific CSF-biomarker profile may be useful for differentiating CAA patients. However, this study was limited by analysing non-individual participant data from heterogeneous cohorts, including also patients with acute intracerebral or subarachnoid haemorrhages, which overall did not allow adjustments for relevant confounding. The reported biomarker profile was later supported in an unadjusted analysis of a larger cohort of 63 patients with CAA,^11^ whereas another analysis of 31 CAA patients contrarily documented that amyloid biomarkers were not helpful in distinguishing CAA from Alzheimer’s disease patients.^12^ In turn, another small study (*n* = 10) was able to replicate the results of reduced Aβ-40 and Aβ-42 levels in CAA patients also in age-adjusted analyses.^31^

This study used a large sample (*n* = 372) allowing comparisons across various diseases and applying important adjustments for multiple confounders, and thus for the first time comprehensively validated a CSF profile in CAA. As CAA mostly causes mild cognitive deficits^32^ we expanded our comparator groups, beyond Alzheimer’s disease and HC, including also participants with MCI and MCI due to Alzheimer’s disease. We here document a specific and independent CSF amyloid pattern in CAA patients with significantly decreased Aβ-40 levels compared across all other diseases and significantly decreased Aβ-42 compared to non-Alzheimer’s disease patients. Evaluating patients with possible versus probable CAA provided equally decreased Aß40 levels in both groups. When considering the full spectrum of NDD biomarkers in CSF using hierarchical cluster analysis, we found that patients with CAA who formed a superordinate group did not differ significantly in the major structural MRI markers, i.e. cerebral micro-haemorrhages or superficial sideroses, compared with those who did not. However, there were two findings more frequently observed in patients within the CAA cluster, i.e. cerebral white matter changes and prior cerebral ischaemia, theoretically hinting towards decreased vascular reactivity with consecutive hypoxia in these patients.^33^ Little is known about the time course of disease progression (specific MRI changes, ischaemic versus haemorrhagic lesions) in CAA, which deserves further future investigations. We believe that our results support the hypothesis that CSF-NDD-biomarkers may be particularly useful for early and independent detection of CAA; possibly comparable to CSF changes detectable years in advance before actual diagnosis of Alzheimer’s disease.^34^

We identified and confirmed several independent clinical associations with CAA, e.g. age and prior lobar ICH are known factors associated with CAA and constitute variables of the MRI-based modified Boston criteria.^4^ Ischaemic brain injury is increasingly recognized to be mediated by CAA and may be induced by hypoperfusion and ischaemia around the amyloid-laden and impaired neurovascular units.^33^ The exact pathophysiological mechanism causing TFNE remains unknown, and hypotheses include cortical spreading depolarization, focal seizures and vasospasm/hypoperfusion, all triggered by blood breakdown products.^16,35^ Further, TFNEs are associated with a higher burden of structural CAA-related damage, specifically involving cortical structures (e.g. cortical superficial siderosis), and clinically important with a higher risk for new or recurrent lobar ICH.^36-39^ Limited data is available regarding mechanisms leading to gait disturbance,^40^ but inferring to a more global perspective of CAA related brain damage, structural and functional network connections may play a pivotal role not only in the development of gait disturbance but also in progression of dementia, cognitive decline and mood disorder.^3,41^

Integrating a multiparametric work-up for CAA diagnosis with CSF-NDD-biomarkers and clinical characteristics along with imaging and genetic examination into diagnostics may be particularly helpful for correct and potentially early diagnosis. This aspect is gaining greater importance, as demonstrated by the recent approval of the anti-amyloid β monoclonal antibodies aducanumab and lecanemab by the US Food & Drug Administration for the treatment of Alzheimer’s disease. According to the FDA, the safety of aducanumab has not been established in patients with any localized superficial siderosis, 10 or more cerebral microhaemorrhages (CMB), and/or with macrohaemorrhages—all of which are imaging findings clearly associated with CAA.^42^ Experts in the field explicitly discourage the use of amyloid antibodies in CAA patients, based on the suggested increased risk for amyloid-related imaging abnormalities, manifesting as oedema or hemosiderin depositions.^43^ Given the frequent pathological overlap of 60% to almost 100% of CAA with Alzheimer’s disease and known correlations for both incidence and severity,^44,45^ an improved identification of patients with CAA might be valuable to increase the safety for these drugs with a debated risk-cost-benefit balance.^46^

Beyond several strengths of this study, our results should be understood within the context of the limitations pertaining to observational data. Diagnosis of CAA was MRI-based by raters blinded to clinical or CSF data using the modified Boston criteria without pathological confirmation. Originally, the Boston criteria, and predominantly their modification, were developed in patients with lobar ICH.^47,48^ Yet, validation studies confirmed that MRI-based diagnosis of probable CAA was associated with moderate or severe CAA among pathological examination in almost 90% of patients even without lobar macro-haemorrhage.^49^ The retrospective design may have attenuated data quality as we included only patients with CSF-biomarkers and blood-sensitive MR imaging simultaneously available. We therefore may have missed patients with CAA and admission for acute stroke, either ischaemic or haemorrhagic, with both diseases potentially falsifying acute CSF diagnostics. In addition, prodromal or asymptomatic patients, as well as patients not eligible for CSF or MRI diagnosis, might have been missed. Therefore, selection bias may be present, yet we believe that our approach represents a real-world diagnostic scenario that increases the generalizability of our results to patients who are actually being evaluated for cognitive complaints. Furthermore, this is the first analysis of CSF-biomarkers using statistical methodologies to adjust for potential confounding by varying inter-group characteristics. During the 10-year-inclusion interval, laboratory assays to measure the CSF-biomarkers were changed for commercial reasons. In order to assure the transition of a diagnostically relevant interpretation, we have adjusted the laboratory protocols and the reference values pending strictly monitored ISO 15 189 Norm and integrated this confounding variable into statistical adjustment. In light of potential interlaboratory differences in CSF amyloid levels, we further provided assay-adjusted mean differences between investigated diseases and controls to ease the translation of results to differing settings.

In summary, patients with CAA showed independent associations with specific clinical- and CSF-biomarkers when compared to patients with Alzheimer’s disease, MCI with or without underlying Alzheimer’s disease, and HC. Our findings may aid clinical decision-making, allocation of advanced diagnostics, and supports a multiparametric approach for the diagnosis of CAA among patients with cognitive complaints. However, validation with improved study design including prospective data collection and pathological confirmation seems warranted.

## Supplementary material

Supplementary material is available at Brain Communications online.

## Supplementary Material

fcad159_Supplementary_Data

## Data Availability

Data that support the study’s findings are available from the corresponding author upon reasonable request and after approval of the data coordinating center.

## Abbreviations

Aβ =: β-amyloid
AUROC =: area under the receiver operating characteristic curve
CAA =: cerebral amyloid angiopathy
CI =: confidence intervals
CSF =: cerebrospinal fluid
DoM =: differences of means
HC =: healthy controls
ICH =: intracerebral haemorrhage
IQR =: interquartile range
MCI =: mild cognitive impairment
NDD =: neurochemical dementia diagnostic
OR =: odds ratio
ROC =: receiver operating characteristic
SD =: standard deviations
TFNE =: transient focal neurologic episodes
