# Supplementary material for: Cerebrospinal fluid biomarkers for cerebral amyloid angiopathy
Source: Brain Commun. 2023 May 19;5(3):fcad159. doi: 10.1093/braincomms/fcad159 (PMC10300526; doi:10.1093/braincomms/fcad159)
Supplement: fcad159_Supplementary_Data [file fcad159_supplementary_data.docx]

**Supplementary material**

*Sembill et al.* Cerebrospinal fluid biomarkers for cerebral amyloid angiopathy

Content:

**Supplemental Table 1.**

Inter-group comparison of patient characteristics (full table)

**Supplemental Table 2.**

Post-hoc tests on patient characteristics with relevant inter-group differences (p<0.1)

**Supplemental Figure 1.**

Imaging of two included patients with likely cerebral amyloid angiopathy despite borderline findings according to modified Boston criteria

**Supplemental Figure 2.**

Inter-group comparison of Aβ40/Aβ42-ratio

**Supplemental Table 3.**

Comparison of CSF levels between groups

**Supplemental Table 4.**

Comparison of CSF levels in patients with probable CAA vs. possible CAA

**Supplemental Table 5.**

Sensitivity analyses of patients with CAA compared to pooled controls

**Supplemental Table 6.**

Multivariate modeling for association with CAA including β-amyloid 40

**Supplemental Table 7.**

Multivariate modeling for association with CAA including β-amyloid 42

**Supplemental Figure 3.**

Adjusted core cerebrospinal fluid biomarker levels

**Supplemental Table 8.**

Inter-group comparison of adjusted cerebrospinal fluid levels

**Supplemental Table 9.**

Post-hoc tests on adjusted cerebrospinal fluid levels

**Supplemental Figure 4.**

Adjusted receiver operating characteristic curves to discriminate between patients with CAA and Alzheimer's disease or healthy controls

**Supplemental Table 10.**

MRI characteristics of patients with CAA in relation to hierarchical cluster analysis

**Supplemental Table 1.** Inter-group comparison of patient characteristics (full table)

| Characteristics | CAA  (n=67) | Probable AD  (n=76) | MCI due to AD  (n=75) | MCI  (n=76) | HC  (n=78) | p-value |
| --- | --- | --- | --- | --- | --- | --- |
| Age, years, median (IQR) | 75 (68-79) | 72 (60-77) | 72 (65-75) | 68 (57-74) | 58 (54-65) | <0.001 |
| Sex, female, n (%) | 27 (40.3%) | 39 (51.3%) | 37 (49.3%) | 33 (43.4%) | 30 (38.5%) | 0.43 |
| Level of education (n=326) |  |  |  |  |  |  |
| Low, n (%) | 15/55 (27.3%) | 24/61 (39.3%) | 22/66 (33.3%) | 26/71 (36.6%) | 22/73 (30.1%) | 0.61 |
| Medium, n (%) | 22/55 (40.0%) | 26/61 (42.6%) | 24/66 (36.4%) | 22/71 (31.0%) | 28/73 (38.4%) |  |
| High, n (%) | 18/55 (32.7%) | 11/61 (18.0%) | 20/66 (30.3%) | 23/71 (32.4%) | 23/73 (31.5%) |  |
| Prior medical history |  |  |  |  |  |  |
| Intracerebral hemorrhage, n (%) | 11 (16.4%) | 0 (0.0%) | 1 (1.3%) | 2 (2.6%) | 1 (1.3%) | <0.001 |
| Lobar, n (%) | 9 (13.4%) | 0 (0.0%) | 1 (1.3%) | 1 (1.3%) | 1 (1.3%) | <0.001 |
| Deep, n (%) | 2 (3.0%) * | 0 (0.0%) | 0 (0.0%) | 1 (1.3%) | 0 (0.0%) | 0.10 |
| Ischemic stroke, n (%) | 19 (28.4%) | 9 (11.8%) | 1 (1.3%) | 13 (17.1%) | 7 (9.0%) | <0.001 |
| Transient ischemic attack, n (%) | 3 (4.5%) | 5 (6.6%) | 0 (0.0%) | 2 (2.6%) | 3 (3.8%) | 0.20 |
| Subarachnoid hemorrhage, n (%) | 2 (3.0%) | 0 (0.0%) | 0 (0.0%) | 2 (2.6%) | 0 (0.0%) | 0.16 |
| Arterial hypertension, n (%) | 56 (83.6%) | 51 (67.1%) | 48 (64.0%) | 53 (69.7%) | 49 (62.8%) | 0.06 |
| Diabetes mellitus, n (%) | 17 (25.4%) | 8 (10.5%) | 10 (13.3%) | 9 (11.8%) | 11 (14.1%) | 0.10 |
| Coronary artery disease, n (%) | 9 (13.4%) | 11 (14.5%) | 10 (13.3%) | 8 (10.5%) | 5 (6.4%) | 0.53 |
| Congestive heart failure, n (%) | 7 (10.4%) | 7 (9.2%) | 5 (6.7%) | 8 (10.5%) | 2 (2.6%) | 0.31 |
| Chronic kidney disease, n (%) | 7 (10.4%) | 7 (9.2%) | 7 (9.3%) | 6 (7.9%) | 4 (5.1%) | 0.80 |
| History of smoking, n (%) | 17 (25.4%) | 24 (31.6%) | 16 (21.3%) | 20 (26.3%) | 23 (29.5%) | 0.57 |
| History of alcohol use, n (%) | 3 (4.5%) | 4 (5.3%) | 3 (4.0%) | 3 (3.9%) | 3 (3.8%) | 0.99 |
| Family history of dementia, n (%) (n=324) | 12/44 (27.3%) | 20/70 (28.6%) | 28/66 (42.4%) | 14/67 (20.9%) | 17/77 (22.1%) | 0.04 |
| Prior medication |  |  |  |  |  |  |
| Antiplatelet therapy, n (%) | 27 (40.3%) | 22 (28.9%) | 29 (38.7%) | 23 (30.3%) | 19 (24.4%) | 0.19 |
| Oral anticoagulants, n (%) | 5 (7.5%) | 6 (7.9%) | 1 (1.3%) | 5 (6.6%) | 1 (1.3%) | 0.10 |
| Statins, n (%) | 23 (34.3%) | 23 (30.3%) | 28 (37.3%) | 22 (28.9%) | 15 (19.2%) | 0.14 |
| Clinical features |  |  |  |  |  |  |
| Aggressive behaviour, n (%) | 2 (3.0%) | 5 (6.6%) | 2 (2.7%) | 0 (0.0%) | 0 (0.0%) | 0.04 |
| Apraxia, n (%) | 4 (6.0%) | 10 (13.2%) | 1 (1.3%) | 1 (1.3%) | 0 (0.0%) | <0.001 |
| Vertigo, n (%) | 3 (4.5%) | 2 (2.6%) | 2 (2.7%) | 1 (1.3%) | 1 (1.3%) | 0.72 |
| Gait disturbance, n (%) | 16 (23.9%) | 5 (6.6%) | 3 (4.0%) | 8 (10.5%) | 1 (1.3%) | <0.001 |
| Recurrent falls, n (%) | 9 (13.4%) | 3 (3.9%) | 3 (4.0%) | 8 (10.5%) | 4 (5.1%) | 0.09 |
| Transient focal neurologic episodes, n (%) | 8 (11.9%) | 4 (5.3%) | 0 (0.0%) | 2 (2.6%) | 2 (2.6%) | <0.01 |
| Predominantly positive symptoms, n (%) | 5 (7.5%) | 0 (0.0%) | 0 (0.0%) | 0 (0.0%) | 1 (1.3%) | 0.001 |
| Predominantly negative symptoms, n (%) | 3 (4.5%) | 4 (5.3%) | 0 (0.0%) | 2 (2.6%) | 2 (2.6%) | 0.32 |
| Duration, minutes | 5.5 (±5.2) | 103.3 (±121.0) | N/A | 180 (±0.0) | 100.0 (±113.1) | 0.15 |
| Repetitive, n (%) | 6 (9.0%) | 0 (0.0%) | 0 (0.0%) | 0 (0.0%) | 0 (0.0%) | <0.001 |
| Stereotypical, n (%) | 5 (7.5%) | 0 (0.0%) | 0 (0.0%) | 0 (0.0%) | 0 (0.0%) | <0.001 |

Compared by Kruskal Wallis H-Test, Pearson's chi-squared test, or the Freeman-Halton extension of the Fisher’s exact test. Abbreviations: Alzheimer’s disease, AD; Cerebral amyloid angiopathy, CAA; Confidence Intervals, CI; Healthy controls, HC; Interquartile range, IQR; Mild cognitive impairment; MCI.

* Two patients with a history of single deep ICH were nevertheless counted as possible CAA because of high lobar microbleed burden. For neuroradiologic details of these two patients, see supplemental Figure 1. The main results regarding CSF biomarkers did not change after exclusion of these two patients (data not shown)**.**

**Supplemental Table 2.** Post-hoc tests on patient characteristics with relevant inter-group differences (p<0.1)

| **Parameters** | ***p*-value for post-hoc tests** | | | | | | | | | |
| --- | --- | --- | --- | --- | --- | --- | --- | --- | --- | --- |
|  | CAA  vs AD | CAA  vs AD-MCI | CAA  vs MCI | CAA  vs HC | AD  vs AD-MCI | AD  vs MCI | AD  vs HC | AD-MCI  vs MCI | AD-MCI  vs HC | MCI  vs HC |
| Age | 0.050 | **0.004** | **<0.001** | **<0.001** | 0.708 | **0.010** | **<0.001** | **0.011** | **<0.001** | **<0.001** |
| Intracerebral hemorrhage | **<0.001** | **0.001** | **0.004** | **0.001** | 0.497 | 0.497 | 1.000 | 1.000 | 1.000 | 0.618 |
| Lobar | **0.001** | **0.006** | **0.006** | **0.006** | 0.497 | 1.000 | 1.000 | 1.000 | 1.000 | 1.000 |
| Deep | 0.218 | 0.221 | 0.600 | 0.212 | 1.000 | 1.000 | 1.000 | 1.000 | 1.000 | 0.494 |
| Prior ischemic stroke | **0.013** | **<0.001** | 0.107 | **0.002** | **0.018** | 0.356 | 0.560 | **0.001** | 0.064 | 0.133 |
| Arterial hypertension | **0.023** | **0.009** | 0.052 | **0.005** | 0.688 | 0.727 | 0.577 | 0.454 | 0.880 | 0.364 |
| Diabetes mellitus | **0.020** | 0.068 | **0.036** | 0.087 | 0.595 | 0.797 | 0.500 | 0.782 | 0.890 | 0.677 |
| Family history of dementia | 0.881 | 0.106 | 0.438 | 0.520 | 0.091 | 0.298 | 0.365 | **0.008** | **0.009** | 0.863 |
| Oral anticoagulants | 0.923 | 0.100 | 1.000 | 0.095 | 0.116 | 0.754 | 0.062 | 0.209 | 1.000 | 0.114 |
| Aggressive behaviour | 0.448 | 1.000 | 0.218 | 0.212 | 0.442 | 0.058 | **0.027** | 0.245 | 0.239 | 1.000 |
| Apraxia | 0.149 | 0.188 | 0.186 | **0.043** | **0.005** | **0.005** | **0.001** | 1.000 | 0.490 | 0.494 |
| Gait disturbance | **0.004** | **0.001** | **0.033** | **<0.001** | 0.719 | 0.384 | 0.114 | 0.123 | 0.360 | **0.017** |
| Recurrent falls | **0.041** | **0.044** | 0.592 | 0.081 | 1.000 | 0.118 | 1.000 | 0.123 | 1.000 | 0.211 |
| Transient focal neurologic episodes | 0.151 | **0.002** | **0.046** | **0.044** | 0.120 | 0.681 | 0.439 | 0.497 | 0.497 | 1.000 |
| Predominantly positive symptoms | **0.021** | **0.022** | **0.021** | 0.095 | 1.000 | 1.000 | 1.000 | 1.000 | 1.000 | 1.000 |

Post-hoc tests were computed using the Mann-Whitney U test, Student's t-test, Pearson’s chi-squared test or the Fisher’s exact test, as appropriate (significant values in bold).

Abbreviations: Alzheimer’s disease, AD; Cerebral amyloid angiopathy, CAA; Healthy controls, HC; Mild cognitive impairment; MCI; Mild cognitive impairment due to Alzheimer’s disease, AD-MCI.

**Supplemental Figure 1.** Imaging of two included patients with likely cerebral amyloid angiopathy despite borderline findings according to modified Boston criteria


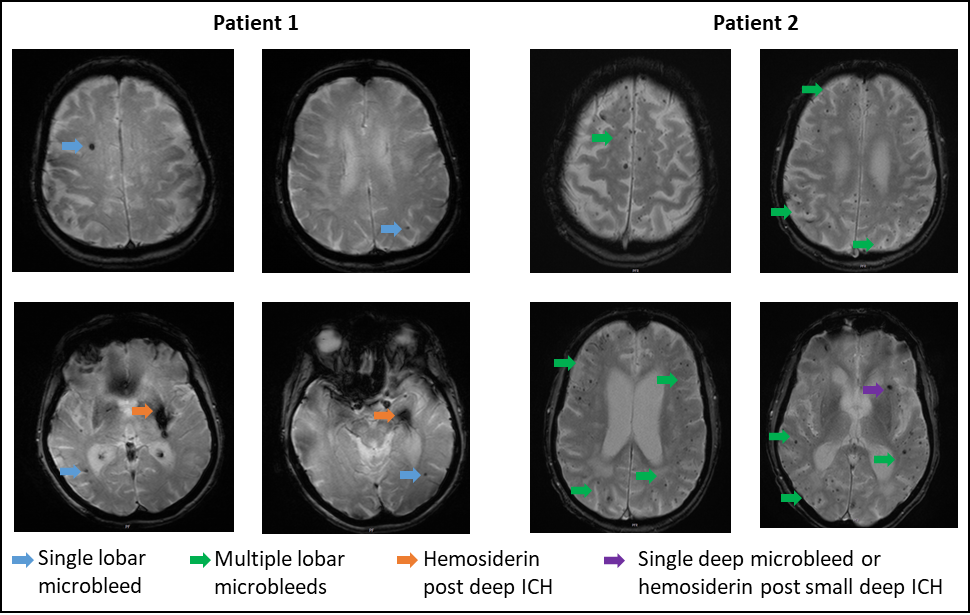


MRI of patient 1 shows multiple lobar microhemorrhages and hemosiderin deposition after macrohemorrhage in the border area between temporal lobe and basal ganglia/thalamus. MRI of patient 2 shows a large number of strictly lobar microhemorrhages and a single deep microhemorrhage or hemosiderin deposition following a clinically pre-documented macrohemorrhage in this area. Therefore, we classified these two patients as possible CAA because at least a CAA copathology seems likely in these cases.

**Supplemental Figure 2.** Inter-group comparison of Aβ40/Aβ42-ratio


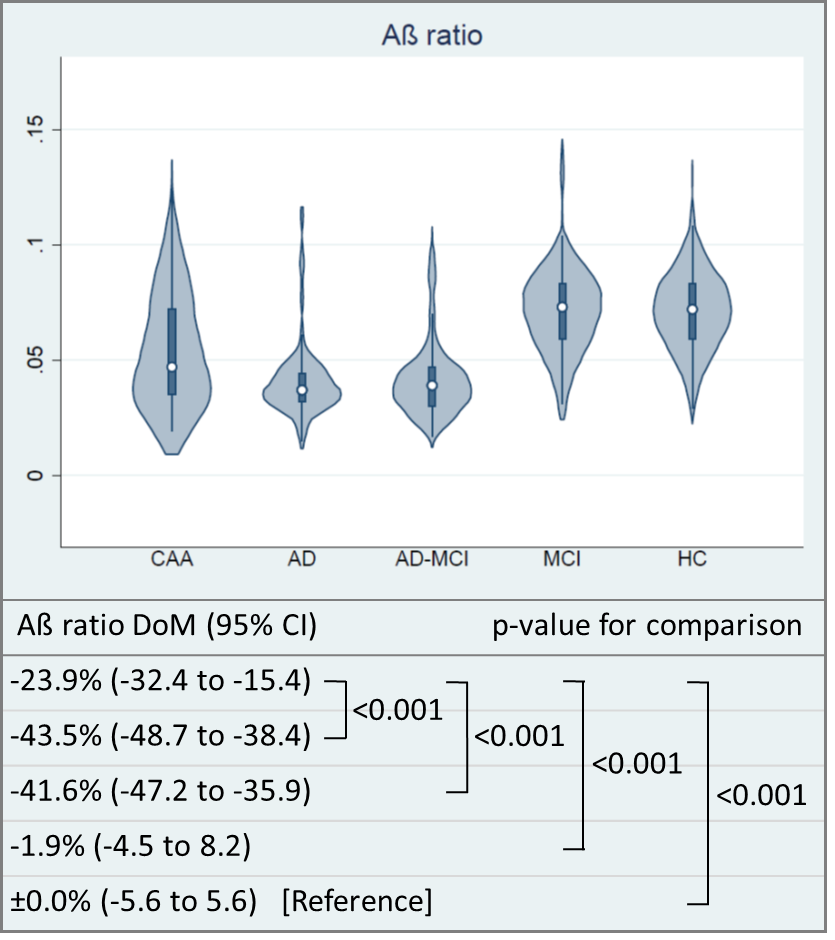


Inter-group comparison of Aβ40/Aβ42 levels using violin plots and assay-specific Differences of Means (DoM) with 95% Confidence Intervals (CI) in relation to HC as a reference. DoM of patients with CAA were compared to all controls by Mann-Whitney U-test.

Abbreviations: Alzheimer’s disease, AD; β-amyloid, Aβ; Cerebral amyloid angiopathy, CAA; Healthy controls, HC; Mild cognitive impairment; MCI; Mild cognitive impairment due to Alzheimer’s disease, AD-MCI.

**Supplemental Table 3.** Comparison of CSF levels between groups

|  | ***p*-value for comparison** | | | | | | | | | |
| --- | --- | --- | --- | --- | --- | --- | --- | --- | --- | --- |
|  | CAA  vs AD | CAA  vs AD-MCI | CAA  vs MCI | CAA  vs HC | AD  vs AD-MCI | AD  vs MCI | AD  vs HC | AD-MCI  vs MCI | AD-MCI  vs HC | MCI  vs HC |
| Aß 40 | **0.023** | **0.001** | **0.023** | **0.004** | 0.265 | 0.916 | 0.454 | 0.327 | 0.750 | 0.522 |
| Aß 42 | 0.102 | 0.932 | **<0.001** | **<0.001** | **0.044** | **<0.001** | **<0.001** | **<0.001** | **<0.001** | 0.509 |
| Aß ratio | **0.001** | **0.004** | **<0.001** | **<0.001** | 0.664 | **<0.001** | **<0.001** | **<0.001** | **<0.001** | 0.882 |
| p-Tau | **<0.001** | **0.001** | **<0.001** | **<0.001** | 0.555 | **<0.001** | **<0.001** | **<0.001** | **<0.001** | 0.964 |
| t-Tau | **0.001** | 0.074 | **<0.001** | **<0.001** | 0.076 | **<0.001** | **<0.001** | **<0.001** | **<0.001** | 0.357 |

Comparison of cerebrospinal fluid (CSF) levels were computed using the Mann-Whitney U test (significant values in bold).

Abbreviations: Alzheimer’s disease, AD; ; β-amyloid, Aβ; Cerebral amyloid angiopathy, CAA; Healthy controls, HC; Mild cognitive impairment; MCI; Mild cognitive impairment due to Alzheimer’s disease, AD-MCI.

**Supplemental Table 4.** Comparison of CSF levels in patients with possible CAA vs. probable CAA

| **Parameters** | **Possible CAA (n=38)** | **Probable CAA (n=29)** | ***p*-value** |
| --- | --- | --- | --- |
| Aß 40, pg/ml | 13,381 (9,598-17,462) | 14,760 (10,608-18,623) | 0.46 |
| Aß 42, pg/ml | 704 (515-885) | 610 (435-728) | 0.09 |
| Aß ratio | 0.059 (0.042-0.079) | 0.041 (0.028-0.063) | 0.02 |
| p-Tau, pg/ml | 64.1 (42.4-90.3) | 68.2 (44.0-99.9) | 0.60 |
| t-Tau, pg/ml | 475 (264-713) | 486 (285-657) | 0.78 |

Intra-group comparison of cerebrospinal fluid (CSF) levels of CAA patients with possible vs. probable CAA using the Mann-Whitney U test.

Abbreviations: β-amyloid, Aβ; Cerebral amyloid angiopathy, CAA.

**Supplemental Table 5.** Sensitivity analyses of patients with CAA compared to pooled controls

| **Characteristics** | **CAA**  **(n=67)** | **No-CAA**  **(n=305)** | **Absolute risk difference**  **(95% CI)** | **Standardized mean difference** |
| --- | --- | --- | --- | --- |
| Age, years, median (IQR) | 75 (68-79) | 67 (58-75) | 8.0 (3.0-13.0) | 0.76 |
| Sex, female, n (%) | 27 (40.3%) | 139 (45.6%) | -5.3 (-18.3 to 7.7) | -0.11 |
| Prior medical history |  |  |  |  |
| Intracerebral hemorrhage, n (%) | 11 (16.4%) | 4 (1.3%) | 15.1 (6.1 to 24.1) | 0.55 |
| Lobar, n (%) | 9 (13.4%) | 3 (1.0%) | 12.4 (4.2 to 20.7) | 0.49 |
| Deep, n (%) | 2 (3.0%) | 1 (0.3%) | 2.7 (-1.5 to 6.8) | 0.21 |
| Ischemic stroke, n (%) | 19 (28.4%) | 31 (10.2%) | 18.2 (6.9 to 29.5) | 0.47 |
| Transient ischemic attack, n (%) | 3 (4.5%) | 10 (3.3%) | 1.2 (-4.1 to 6.5) | 0.06 |
| Subarachnoid hemorrhage, n (%) | 2 (3.0%) | 2 (0.7%) | 2.3 (-1.8 to 6.5) | 0.17 |
| Arterial hypertension, n (%) | 56 (83.6%) | 201 (65.9%) | 17.7 (7.3 to 28.0) | 0.41 |
| Diabetes mellitus, n (%) | 17 (25.4%) | 38 (12.5%) | 12.9 (1.9 to 24.0) | 0.33 |
| Coronary artery disease, n (%) | 9 (13.4%) | 34 (11.2%) | 2.3 (-6.6 to 11.2) | 0.07 |
| Congestive heart failure, n (%) | 7 (10.4%) | 22 (7.2%) | 3.2 (-4.6 to 11.1) | 0.11 |
| Chronic kidney disease, n (%) | 7 (10.4%) | 24 (7.9%) | 2.6 (-5.3 to 10.5) | 0.09 |
| History of smoking, n (%) | 17 (25.4%) | 83 (27.2%) | -1.9 (-13.8 to 9.9) | -0.04 |
| History of alcohol use, n (%) | 3 (4.5%) | 13 (4.3%) | 0.2 (-5.4 to 5.8) | 0.01 |
| Family history of dementia, n (%) (n=324) | 12/44 (27.3%) | 79/280 (28.3%) | -0.9 (-15.1 to 13.2) | -0.02 |
| Prior medication |  |  |  |  |
| Antiplatelet therapy, n (%) | 27 (40.3%) | 93 (30.5%) | 9.8 (-3.0 to 22.6) | 0.21 |
| Oral anticoagulants, n (%) | 5 (7.5%) | 13 (4.3%) | 3.2 (-3.5 to 9.9) | 0.14 |
| Statins, n (%) | 23 (34.3%) | 88 (28.9%) | 5.5 (-7.0 to 17.9) | 0.12 |
| Clinical features |  |  |  |  |
| Aggressive behaviour, n (%) | 2 (3.0%) | 7 (2.3%) | 0.7 (-3.7 to 5.1) | 0.04 |
| Apraxia, n (%) | 4 (6.0%) | 12 (3.9%) | 2.0 (-4.0 to 8.1) | 0.09 |
| Vertigo, n (%) | 3 (4.5%) | 6 (2.0%) | 2.5 (-2.7 to 7.7) | 0.14 |
| Gait disturbance, n (%) | 16 (23.9%) | 17 (5.6%) | 18.3 (7.8 to 28.8) | 0.53 |
| Recurrent falls, n (%) | 9 (13.4%) | 18 (5.9%) | 7.5 (-1.1 to 16.1) | 0.26 |
| Transient focal neurologic episodes, n (%) | 8 (11.9%) | 8 (2.6%) | 9.3 (1.3 to 17.3) | 0.36 |
| Core cerebrospinal fluid levels |  |  |  |  |
| Aß 40, pg/ml, median (IQR) | 13,792 (10,081-18,063) | 16,303 (12,470-20,847) | -2,470 (-4,487 to -453) | -0.45 |
| Aß 42, pg/ml, median (IQR) | 634 (492-834) | 802 (581-1,176) | -168 (-313 to -23) | -0.45 |
| p-Tau, pg/ml, median (IQR) | 67.3 (42.9-91.9) | 63.0 (44.1-88.7) | 4.3 (-7.7 to 16.3) | -0.10 |
| t-Tau, pg/ml, median (IQR) | 468 (275-698) | 362 (241-589) | 122 (27 to 216) | 0.21 |

Abbreviations: β-amyloid, Aβ; Cerebral amyloid angiopathy, CAA; Confidence Intervals, CI; Interquartile range, IQR.

**Supplemental Table 6**.

Multivariate modeling for association with CAA including β-amyloid 40

| **Parameter** | **Odds ratio (95% CI)** | **p-value** |
| --- | --- | --- |
| Age, years | 1.06 (1.02-1.10) | <0.01 |
| Arterial hypertension | 1.56 (0.67-3.64) | 0.30 |
| Diabetes mellitus | 1.31 (0.60-2.87) | 0.50 |
| Prior lobar ICH | 8.46 (1.50-47.72) | 0.02 |
| Prior ischemic stroke | 3.04 (1.43-6.46) | <0.01 |
| Transient focal neurological episodes | 4.19 (1.06-16.64) | 0.04 |
| Gait disturbance | 2.24 (0.90-5.59) | 0.08 |
| t-Tau, pg/ml | 1.0007 (0.9998-1.0017) | 0.14 |
| β-amyloid 40, pg/ml | 0.9999 (0.9998-1.0000) | <0.01 |

The multivariate model included parameters demonstrating significance in sensitivity analyses of CAA patients compared to pooled controls.

Abbreviations: Cerebral amyloid angiopathy, CAA; Confidence Intervals, CI; Intracerebral hemorrhage, ICH.

**Supplemental Table 7**.

Multivariate modeling for association with CAA including β-amyloid 42

| **Parameter** | **Odds ratio (95% CI)** | **p-value** |
| --- | --- | --- |
| Age, years | 1.06 (1.02-1.10) | <0.01 |
| Arterial hypertension | 1.52 (0.66-3.50) | 0.33 |
| Diabetes mellitus | 1.40 (0.64-3.06) | 0.40 |
| Prior lobar ICH | 14.00 (2.64-74.19) | <0.01 |
| Prior ischemic stroke | 3.36 (1.58-7.11) | <0.01 |
| Transient focal neurological episodes | 3.55 (0.95-13.32) | 0.06 |
| Gait disturbance | 2.82 (1.11-7.15) | 0.03 |
| t-Tau, pg/ml | 1.0002 (0.9993-1.0011) | 0.71 |
| β-amyloid 42, pg/ml | 0.9989 (0.9980-0.9998) | 0.01 |

The multivariate model included parameters demonstrating significance in sensitivity analyses of CAA patients compared to pooled controls.

Abbreviations: Cerebral amyloid angiopathy, CAA; Confidence Intervals, CI; Intracerebral hemorrhage, ICH.

**Supplemental Figure 3.** Adjusted core cerebrospinal fluid biomarker levels


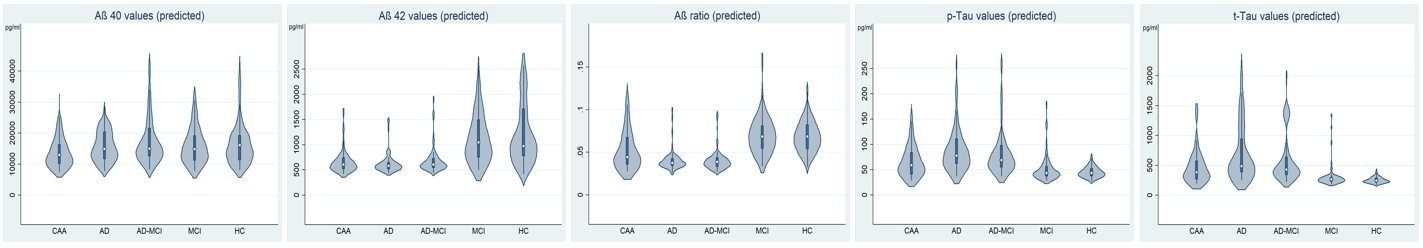


Adjusted core cerebrospinal fluid biomarkers are graphically compared using violin plots; adjusted for age, arterial hypertension, diabetes mellitus, prior lobar intracerebral hemorrhage, prior ischemic stroke, transient focal neurological episodes and gait disturbance.

Abbreviations: Alzheimer’s disease, AD; β-amyloid, Aβ; Cerebral amyloid angiopathy, CAA; Healthy controls, HC; Mild cognitive impairment; MCI; Mild cognitive impairment due to Alzheimer’s disease, AD-MCI.

**Supplemental Table 8.** Inter-group comparison of adjusted cerebrospinal fluid levels

| **Parameters** | **CAA (n=67)** | **AD (n=76)** | **AD-MCI (n=75)** | **MCI (n=76)** | **HC (n=78)** | ***p*-value** |
| --- | --- | --- | --- | --- | --- | --- |
| Aß 40, pg/ml | 13,059 (10,390-17,090) | 15,197 (12,224-20,297) | 15,361 (12,812-21,630) | 15,276 (11,727-19,438) | 16,465 (11,929-20,382) | 0.01 |
| Aß 42, pg/ml | 612 (534-748) | 587 (522-660) | 607 (549-721) | 1,040 (753-1,480) | 1,003 (788-1,767) | <0.001 |
| Aß ratio | 0.043 (0.035-0.070) | 0.037 (0.033-0.042) | 0.038 (0.033-0.043) | 0.069 (0.055-0.082) | 0.069 (0.054-0.083) | <0.001 |
| p-Tau, pg/ml | 60.5 (43.6-84.1) | 78.5 (64.2-109.4) | 72.5 (57.4-102.8) | 45.5 (38.9-58.5) | 45.1 (39.3-53.3) | <0.001 |
| t-Tau, pg/ml | 407 (267-596) | 503 (400-931) | 443 (353-667) | 274 (238-316) | 250 (228-301) | <0.001 |

Cerebrospinal fluid levels were adjusted for age, arterial hypertension, diabetes mellitus, prior lobar intracerebral hemorrhage, prior ischemic stroke, transient focal neurological episodes and gait disturbance using logit regression modeling. Inter-group comparison was performed using the Kruskal-Wallis-test.

Abbreviations: Alzheimer’s disease, AD; β-amyloid, Aβ; Cerebral amyloid angiopathy, CAA; Healthy controls, HC; Mild cognitive impairment; MCI; Mild cognitive impairment due to Alzheimer’s disease, AD-MCI.

**Supplemental Table 9.** Post-hoc tests on adjusted cerebrospinal fluid levels

|  | ***p*-value for post-hoc tests** | | | | | | | | | |
| --- | --- | --- | --- | --- | --- | --- | --- | --- | --- | --- |
|  | CAA  vs AD | CAA  vs AD-MCI | CAA  vs MCI | CAA  vs HC | AD  vs AD-MCI | AD  vs MCI | AD  vs HC | AD-MCI  vs MCI | AD-MCI  vs HC | MCI  vs HC |
| Aß 40 | **0.016** | **0.001** | **0.021** | **0.003** | 0.247 | 0.938 | 0.488 | 0.279 | 0.727 | 0.475 |
| Aß 42 | 0.148 | 0.912 | **<0.001** | **<0.001** | 0.058 | **<0.001** | **<0.001** | **<0.001** | **<0.001** | 0.427 |
| Aß ratio | **0.001** | **0.003** | **<0.001** | **<0.001** | 0.675 | **<0.001** | **<0.001** | **<0.001** | **<0.001** | 0.957 |
| p-Tau | **<0.001** | **0.001** | **<0.001** | **<0.001** | 0.597 | **<0.001** | **<0.001** | **<0.001** | **<0.001** | 0.672 |
| t-Tau | **0.001** | 0.052 | **<0.001** | **<0.001** | 0.094 | **<0.001** | **<0.001** | **<0.001** | **<0.001** | 0.119 |

Post-hoc tests on adjusted cerebrospinal fluid levels were computed using the Mann-Whitney U test (significant values in bold).

Abbreviations: Alzheimer’s disease, AD; ; β-amyloid, Aβ; Cerebral amyloid angiopathy, CAA; Healthy controls, HC; Mild cognitive impairment; MCI; Mild cognitive impairment due to Alzheimer’s disease, AD-MCI.

**Supplemental Figure 4.** Adjusted receiver operating characteristic curves to discriminate between patients with CAA and Alzheimer's disease or healthy controls


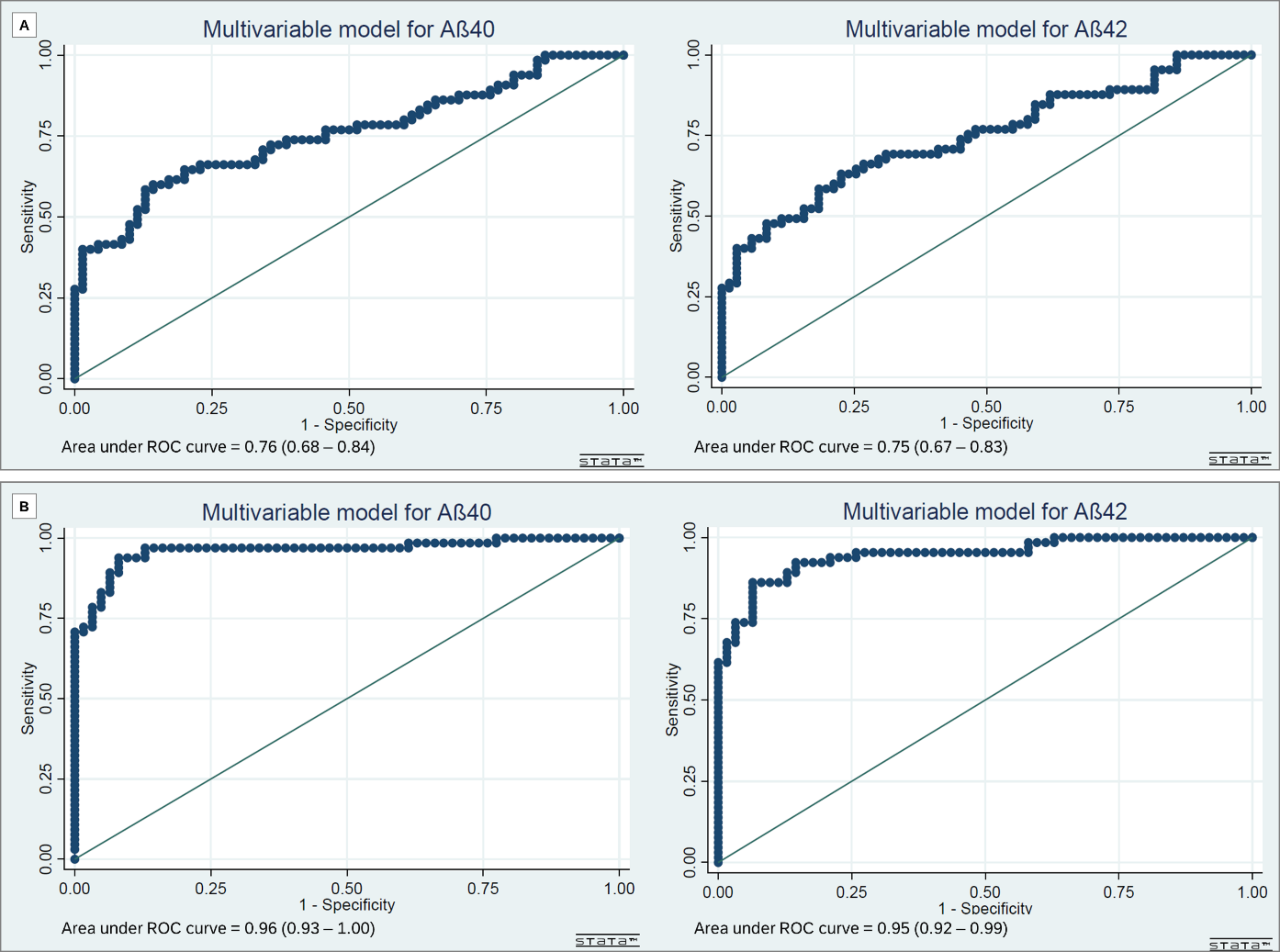


Adjusted receiver operating characteristic (ROC) curves for β-amyloid 40 and β-amyloid 42 based on multivariable modelling including all parameters with high statistical significance (p<0.01) in inter-group comparison, i.e. prior lobar intracerebral hemorrhage, gait disturbance and t-Tau to discriminate (A) patients with cerebral amyloid angiopathy and Alzheimer’s disease; and age, arterial hypertension, prior lobar intracerebral hemorrhage, prior ischemic stroke, gait disturbance and t-Tau to discriminate (B) patients with cerebral amyloid angiopathy and healthy controls.

Abbreviations: β-amyloid, Aβ.

**Supplemental Table 10.**

MRI characteristics of patients with CAA in relation to hierarchical cluster analysis

| **Parameters** | **No CAA cluster (n=36)** | **CAA cluster (n=31)** | **p-value** |
| --- | --- | --- | --- |
| Cerebral microbleeds |  |  |  |
| Lobar, n (%) | 34 (94.4%) | 26 (83.9%) | 0.24 |
| Cerebellar, n (%) | 7 (19.4%) | 9 (29.0%) | 0.36 |
| Total count, median (IQR) | 2 (1-6) | 3 (1-24) | 0.53 |
| Superficial siderosis, n (%) | 8 (22.2%) | 6 (19.4%) | 0.78 |
| Focal, n (%) | 6 (16.7%) | 4 (12.9%) | 0.74 |
| Disseminated, n (%) | 2 (5.6%) | 2 (6.5%) | 1.00 |
| Prior cerebral ischemia, n (%) | 5 (13.9%) | 11 (35.5%) | 0.04 |
| Lacunar infarction, n (%) | 5 (13.9%) | 9 (29.0%) | 0.13 |
| Territorial infarction, n (%) | 1 (2.8%) | 3 (9.7%) | 0.33 |
| Prior intracerebral hemorrhage, n (%) | 3 (8.3%) | 5 (16.1%) | 0.46 |
| Lobar location, n (%) | 3 (8.3%) | 3 (9.7%) | 1.00 |
| Non-lobar location, n (%) | 0 (0.0%) | 2 (6.5%) | 0.21 |
| White matter hyperintensities |  |  |  |
| Fazekas score, median (IQR) | 1 (1-2) | 2 (1-2) | 0.04 |
| Deep |  |  |  |
| Absent, n (%) | 8 (22.2%) | 4 (12.9%) | 0.10 |
| Puncate foci, n (%) | 18 (50%) | 10 (32.3%) |  |
| Beginning confluence, n (%) | 5 (13.9%) | 12 (38.7%) |  |
| Large confluent areas, n (%) | 5 (13.9%) | 5 (16.1%) |  |
| Periventricular |  |  |  |
| Absent, n (%) | 5 (13.9%) | 5 (16.1%) | 0.75 |
| “Caps“ or pencil-thin lining, n (%) | 6 (16.7%) | 7 (22.6%) |  |
| Smooth „halo“, n (%) | 15 (41.7%) | 9 (29.0%) |  |
| Irregular periventricular signal extending  into the deep white matter, n (%) | 10 (27.8%) | 10 (32.3%) |  |
| Anterior |  |  |  |
| Absent, n (%) | 8 (22.2%) | 10 (32.3%) | 0.43 |
| Lucency restricted to region  adjoining ventricles, n (%) | 17 (47.2%) | 10 (32.3%) |  |
| Lucency covering entire region  from lateral ventricle to cortex, n (%) | 11 (30.6%) | 11 (35.5%) |  |
| Posterior |  |  |  |
| Absent, n (%) | 15 (41.7%) | 10 (32.3%) | 0.59 |
| Lucency restricted to region  adjoining ventricles, n (%) | 8 (22.2%) | 6 (19.4%) |  |
| Lucency covering entire region  from lateral ventricle to cortex, n (%) | 13 (36.1%) | 15 (48.4%) |  |
| Modified Boston criteria |  |  |  |
| Probable CAA, n (%) | 15 (41.7%) | 14 (45.2%) | 0.77 |
| Possible CAA, n (%) | 21 (58.3%) | 17 (54.8%) |  |

Compared by Pearson's chi-squared test or Mann-Whitney U test. Abbreviations: Interquartile range, IQR.
